# Evaluation of the paclitaxel–ifosfamide–cisplatin (TIP) combination in relapsed and/or metastatic cervical cancer

**DOI:** 10.1038/sj.bjc.6605305

**Published:** 2009-09-08

**Authors:** C Kosmas, N Mylonakis, G Tsakonas, G Vorgias, N Karvounis, N Tsavaris, T Daladimos, N Kalinoglou, N Malamos, T Akrivos, A Karabelis

**Affiliations:** 1 Second Division of Medical Oncology, Department of Medicine, ‘Metaxa’ Cancer Hospital, Pireaus, Greece; 2 Department of Gynecology, ‘Metaxa’ Cancer Hospital, Pireaus, Greece; 3 Medical Oncology Unit, Department of Pathophysiology-Athens University School of Medicine, Laikon General Hospital, Athens, Greece; 4 Medical Oncology Unit, Department of Medicine, Helena-Venizelou Hospital, Athens, Greece

**Keywords:** paclitaxel, ifosfamide, cisplatin, chemotherapy, cervical cancer

## Abstract

**Background:**

Recurrent or metastatic cervical cancer represents an aggressive malignancy with a high rate of locoregional and distant failure. Therefore, we evaluated the three-drug combination of paclitaxel–ifosfamide–cisplatin (TIP).

**Methods:**

Systemic chemotherapy-naive patients with advanced metastatic/relapsed cervical cancer and a World Health Organization (WHO) performance status (PS) of 0–2 were eligible. TIP chemotherapy doses were paclitaxel 175 mg m^−2^ on day 1, ifosfamide 2.5 g m^−2^ on days 1+2, and cisplatin 40 mg m^−2^ on days 1+2, with prophylactic granulocyte-colony stimulating factor.

**Results:**

A total of 42 patients with recurrent/metastatic cervical cancer are evaluable for response and toxicity: median age: 56 (25–74) years; PS: 1 (0–2); histologies – squamous: 35, adenosquamous: 5, and adenocarcinoma: 2. Responses were overall response rate (RR): 62% (95% confidence interval (CI): 47.3–76.7%), with complete response (CR): 26% (95% CI: 12.7–39.3%), and partial response (PR): 36% (95% CI: 21.5–49.9%). Responses according to the relapse site were overall RR: 32% (95% CI: 13.7–50.3%) within previously irradiated pelvis *vs* 75% (95% CI: 57.7–92.3%) in extra-pelvic sites. Median time to progression (TTP) was 7 (range, 2–34+) months and median overall survival (OS) was 16.5 (range, 3–36+) months. Toxicities included grade 3–4 neutropenia: 83% (21% febrile neutropenia), grade 3–4 thrombocytopenia: 9%, no grade 3 neuropathy (35% grade 2), grade 2 asthenia/fatigue 15%, and no treatment-related deaths.

**Conclusion:**

TIP is an active regimen with acceptable toxicity in advanced/relapsed cervical cancer.

Paclitaxel represents an established active cytotoxic agent against a wide variety of advanced solid tumours, including gynaecological cancers such as ovarian, uterine, and cervical malignancies. Beyond its early documented activity in relapsed ovarian cancer (Eisenhauer *et al*, 1994), there are a small number of studies confirming its activity in advanced endometrial carcinoma (Lissoni *et al*, 1996). Single-agent paclitaxel has shown an initial 17% activity in advanced cervical cancer at a dose of 170 mg m^−2^ in 24-h infusion (Thigpen *et al*, 1995; McGuire *et al*, 1996), whereas subsequent doses of 250 mg m^−2^ over 3 h with granulocyte-colony stimulating factor (G-CSF) support yielded response rates (RRs) of 25% (Kudelka *et al*, 1997). Until now, cisplatin has been the most active cytotoxic drug in advanced cervical cancer, and randomised studies applying combinations of first-generation cytotoxic agents with cisplatin had not shown any advantage over single-agent cisplatin. However, a large randomised study conducted by the Gynecologic Oncology Group (GOG) showed that the cisplatin–ifosfamide combination yielded a 31% RR, significantly higher than the cisplatin–mitolactol combination or cisplatin monotherapy, whereas the median time to progression (TTP) was not prolonged significantly: 4.6 *vs* 3.2 months (Omura *et al*, 1997). Ifosfamide represents another very active antineoplastic agent in advanced/relapsed cervical cancer (Sutton *et al*, 1993). The combination of paclitaxel–cisplatin has yielded very encouraging RRs: 50% in phase II studies (Papadimitriou *et al*, 1999; Rose *et al*, 1999). Moreover, the three-drug combination of paclitaxel–ifosfamide–cisplatin (TIP) has been very active in advanced/metastatic cervical cancer that has relapsed after surgery±radiotherapy (Zanetta *et al*, 1999), whereas it has shown an 84% RR with 16% pathological CR when applied as neoadjuvant chemotherapy before radical surgery in stages IB2–IVA (Zanetta *et al*, 1998).

A phase I study of TIP conducted by our group in various advanced solid tumours has shown the feasibility of administering full doses of each drug with the aid of G-CSF and has established the recommended doses (Kosmas *et al*, 2000a), and subsequent phase II studies in advanced non-small-cell (Kosmas *et al*, 2000b) and small-cell lung cancer (Kosmas *et al*, 2001) have confirmed the feasibility and high anti-tumour activity of the combination.

The aim of this study was to evaluate the feasibility and activity of the TIP regimen in patients with locoregionally relapsed (after radiotherapy±surgery) and/or metastatic cervical cancer.

## Patients and methods

### Patient selection

Consecutive patients with histologically confirmed cervical cancer that had relapsed after previous pelvic radiotherapy±surgery or with distant metastases at presentation, which were referred to three collaborating Medical Oncology units, were candidates for treatment with the TIP chemotherapy regimen. Eligibility included the following: (i) patients aged between 18 and 75 years with histologically confirmed cervical cancer not potentially curable by other local measures such as salvage surgery or radiotherapy; (ii) a World Health Organization (WHO) performance status (PS) of ⩽2; (iii) life expectancy of ⩾3 months; (iv) adequate haematopoietic (ANC >1500 μl^−1^, PLT >100 000 μl^−1^), liver (bilirubin <1.5 mg per 100 ml, AST/ALT <2 × upper normal limit (nl), unless caused by tumour and serum albumin >3.0 g per 100 ml), and renal function (BUN and creatinine <1.5 nl; nl=1.5 mg per 100 ml in our laboratory or creatinine clearance >60 ml min^−1^); (v) no previous systemic chemotherapy (radiosensitising chemotherapy with weekly cisplatin was allowed); (vi) absence of active coronary artery disease (over the last 12 months), unstable diabetes mellitus, or peripheral neuropathy ⩾grade 2 by the WHO criteria; and (vii) presence of bi-dimensionally measurable disease outside a previously irradiated field, unless there was definite evidence of progression at this site. Patients with isolated progression within the pelvis after radical radiotherapy±surgery had to show a ⩾50% increase in the sum of the products of residual lesions at least 3 months after completion of radiotherapy. Patients with brain metastases were excluded from this study. The study was approved by the Institutional Review Board of the participating institutions, and informed consent was obtained from each patient before study entry according to Institutional policies.

### Treatment schedule

Eligible patients were treated as follows: paclitaxel was administered at 175 mg m^−2^ over 1–3 h by intravenous (i.v.) infusion on day 1, after pre-medication consisting of dexamethasone 20 mg, dimethindene maleate (Fenistil) 4 mg, and ranitidine 50 mg, all administered i.v. 1 h before paclitaxel administration (Tsavaris *et al*, 1997). Ifosfamide was administered at 5.0 g m^−2^ i.v. over 1 h divided into 2 days (days 1, 2: 2.5 g m^−2^ per day; total dose 5.0 g m^−2^), together with mesna uroprotection, at 40% of the ifosfamide dose, given i.v., together with ifosfamide infusion, and at 3 and 6 h thereafter. Cisplatin was administered at 80 mg m^−2^ i.v. over 30 min, fractionated for 2 days (days 1, 2: 40 mg m^−2^ per day; total dose 80 mg m^−2^) with adequate vigorous pre- and post-hydration, mannitol and furosemide diuresis, and electrolyte replacement, 20 mequiv. potassium chloride and 8 mequiv. magnesium sulphate per liter of post-hydration solution (0.9% normal saline (N/S) or 1/2 N/S+5% dextrose (D5/w)). The chemotherapy schedule (TIP) was recycled every 21 days.

### Supportive care

Standard anti-emetic medication included ondansetron 24 mg or granisetron 3 mg i.v. 1 h before chemotherapy at 12 h, 8 mg i.v./p.o. or 3 mg i.v./1 mg p.o., respectively, on days 1 and 2 and post-chemotherapy ondasetron 8 mg t.i.d./p.o. or granisetron 1 mg p.o. on days 3–5. Dexamethasone 20 mg i.v. was administered 1 h before chemotherapy (day 1 as paclitaxel pre-medication as well) on days 1 and 2 and post-chemotherapy 4 mg t.i.d. or methylprednisolone 16 mg b.i.d/p.o. on days 3–5 (Kosmas *et al*, 2000a).

Haematopoietic growth factors included G-CSF 5 μg kg^−1^ s.c. (filgrastim or lenograstim) from day 4 until WBC of ⩾10 000 μl^−1^ and recombinant human erythropoietin (rh-Epo) of 10 000 IU s.c./t.i.w or 30,000 IU Epoetin-beta or 40 000 IU Epoetin-alpha × 1/week (not on the days of chemotherapy) whenever the haemoglobin (Hb) value dropped ⩽10.5 g per 100 ml and continued until Hb ⩾12 g per 100 ml.

### Dose modifications for toxicity

The prerequisites for dose modifications were set as follows: (i) any episode of grade 4 neutropenia of >7 days duration, (ii) any episode of febrile ⩾grade 3 neutropenia, (iii) any episode of grade 4 thrombocytopenia, (iv) any non-haematologic grade 3 or 4 toxicity excluding nausea and vomiting, musculoskeletal and arthritic pain (myalgia/arthralgia syndrome), and alopecia.

The following guidelines were applied with respect to dose reductions for toxicity: (i) For neutropenia, meeting the aforementioned criteria, paclitaxel and ifosfamide doses were reduced by 20% in subsequent cycles and if toxicity reappeared after a total of 40% reduction from the starting dose in consecutive cycles, treatment was stopped; however, the patient was evaluable for toxicity and response. (ii) For thrombocytopenia, reduction of cisplatin by 20% was applied in addition to paclitaxel and ifosfamide dose reductions as specified for neutropenia. (iii) For ⩾grade 3 mucositis, the doses of paclitaxel and ifosfamide were reduced by 20% in subsequent cycles. (iv) For neuropathy, ⩾grade 3 treatment was interrupted. (v) For renal toxicity, ⩾3 grade toxicity (serum creatinine elevations >3 × normal) treatment was withheld until recovery (serum creatinine <1.8 mg per 100 ml) with cisplatin and ifosfamide administered with further hydration, mannitol diuresis, and hospitalisation in subsequent cycles. (vi) For ⩾grade 3 CNS toxicity (ifosfamide encephalopathy), the dose of ifosfamide was reduced by 20% and more hydration with bicarbonates was anticipated in subsequent cycles. In the case that encephalopathy reappeared, ifosfamide was omitted from subsequent cycles.

In the case in which blood counts had not recovered to ANC ⩾1.500 μl^−1^ and PLT ⩾100.000 μl^−1^ on the day of therapy, treatment was withheld until recovery, and after a maximum delay of 2 weeks, no further therapy was administered.

### Pretreatment, follow-up studies, and response evaluation

Tumour measurements were determined by physical examination and by the specific radiological test that documented measurable disease before treatment. Before the first chemotherapy cycle, a detailed clinical and gynaecological (pelvic) examination, followed by CT scans of the chest/abdomen/pelvis and radionuclide bone scintigraphy, was carried out in all patients. Computed tomography scans of the brain were carried out in the case of suspected brain metastases. Blood counts were checked weekly after each cycle (days 8 and 15) or more frequently in the case of grade 3–4 haematologic toxicity. Evaluation of response was carried out every three cycles of therapy. Patients experiencing toxic death despite objective responses at measurable sites would be categorised as treatment failures. Complete response (CR) is defined as the disappearance of all signs and symptoms of disease for at least 1 month, with the documented disappearance of all known lesions by physical examination, X-rays, CT scans, bone scans, and the development of no new lesions. Partial response (PR) indicates a decrease of ⩾50%(compared with pretreatment measurements) in the sum of the products of the two largest perpendicular diameters of all measurable lesions and no concomitant growth of new lesions for at least 1 month. There could be no deterioration of symptoms or PS unless secondary to drug toxicity. Stable disease indicates a decrease of <50% or an increase in tumour size <25% over the original measurements. There could be no deterioration of symptoms or PS unless secondary to drug toxicity. Progressive disease (PD) was defined as an increase of ⩾25% over the original measurements.

Full staging evaluation had to be carried out, as reported above, before treatment initiation. Follow-up disease evaluation was carried out at approximately 3-month intervals after the end of treatment.

### Statistical methods

Patients who received at least two cycles of treatment were evaluable for response, unless there was definite evidence of progression after the first cycle for them to be categorised as having PD, and patients who received at least one cycle of treatment were evaluable for toxicity. Response duration was measured from the day of its initial documentation until PD; TTP was calculated from study entry until evidence of PD; OS was measured from the day of entry until last follow-up or death. The 95% confidence intervals (CIs) for RRs were calculated from the binomial distribution (Cox, 1970). Survival was estimated by the product-limit method of Kaplan–Meier (Kaplan and Meier, 1958). The trial was designed as a phase II study, with RR as the main end point. According to Simon's (1989) two-stage design (Simon, 1989), with a sample size of *n*=40, the study has an 80% power to accept the hypothesis that true RR is >50%, and a *P* value <0.05 to reject the hypothesis that the true RR is <30%, if <19 responses occur. At the first stage, if <5 responses occurred out of the initial 16 patients, the study would conclude that the anticipated RR was <30% and terminate, with a power >90%. The study was supervised by the Data Safety Monitoring Board (DSMB) for severe and unacceptable toxicities, and no concern was raised by the investigators and the DSMB regarding safety and efficacy, particularly during the first stage.

## Results

### Patients’ characteristics

Between June 2003 and August 2008, 42 patients with relapsed/metastatic cervical cancer were treated with the TIP chemotherapy regimen. Final data analysis was carried out in March 2009 after all patients entered had completed the planned six cycles of chemotherapy or had interrupted treatment as a result of disease progression or unacceptable toxicity. Patient characteristics and demographics are provided in Table 1. Median age was 56 years (range, 25–74), and 93% had a WHO-PS of 0 or 1, with 7% of patients having a WHO-PS of 2. Overall, 59.5% of patients presented with an advanced FIGO stage III or IV (16.7% with metastatic stage IVB disease). A total of 40.5% of patients had undergone radical hysterectomy and pelvic lymphadenectomy earlier, and 79% had undergone an earlier radical radiotherapy in the pelvis. Overall, 17% of patients had histologies other than squamous carcinoma (12% adenosquamous carcinoma and 5% adenocarcinoma).

### Response to treatment and survival

**Figure 1:**
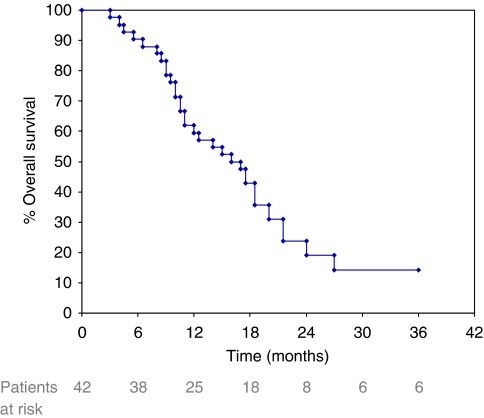
Overall survival data of cervical cancer patients treated with TIP.

Response to TIP chemotherapy was as follows: overall RR: 26 out of 42 (62%, 95% CI: 47.3–76.7%), with CR: 11 out of 42 (26%, 95% CI: 12.7–39.3%) and PR: 15 out of 42 (36%, 95% CI: 21.5–49.9%). Stable disease was observed in 11 out of 42 patients (26%, 95% CI: 12.7–39.3%) and PD in 5 out of 42 patients (12%, 95% CI: 2.2–21.8%). Responses were further subdivided according to disease site, that is, within previously irradiated pelvis *vs* outside pelvis and/or distant sites, and were as follows: overall RR: 8 out of 25 (32%, 95% CI: 13.7–50.3%) in pelvic sites *vs* 18 out of 24 (75%, 95% CI: 57.7–92.3%) (*P*=0.041) in extra-pelvic sites (as seven patients had measurable disease at both irradiated pelvis and extra-pelvic sites) (see also Table 2). Moreover, RR after chemoradiation *vs* others was 15 out of 28 (53.5%, 95% CI: 35–72%) *vs* 11 out of 14 (78.6%, 95% CI: 57.1–100%), respectively (*P*=NS (nonsignificant), as a result of small numbers). The median duration of response (for CR+PR patients) was 6.5 months and median TTP was 7 months (range, 2 to 34+), whereas the median OS was 16.5 months (range, 3 to 36+) (Figure 1).

### Compliance with treatment

A total of 224 treatment cycles (median: 6 cycles; range, 2–6, mean: 5.33 cycles) were administered. Six patients did not complete the planned six cycles as a result of PD detected after the third cycle in five and after the second cycle in one patient. Four more patients did not complete the planned six cycles as a result of: two dose reductions in successive cycles for haematologic toxicity (as defined above) in two patients after the fourth and fifth cycle, and treatment omission for renal toxicity in two patients (after cycles 2 and 4).

### Toxicities

Haematologic and non-haematologic toxicity data for all patients enrolled are summarised in Tables 3 and 4, respectively. Haematologic toxicities (Table 3) consisted primarily of grade 3–4 neutropenia in 83% (57% grade 4) of patients, despite the use of prophylactic G-CSF administration, whereas grade 3–4 thrombocytopenia was encountered in 9% (2% grade 4) of patients. Febrile neutropenia was observed in 9 out of 42 (21%) of patients, with 5 of them developing more than one episode. All febrile neutropenic events were managed successfully in the in-patient or outpatient setting by broad-spectrum antibiotics, and there were no treatment-related deaths.

Non-haematologic toxicities (Table 4) consisted primarily of grade 2–3 nausea and vomiting in 13 out of 22% patients, grade 1–2 myalgia/arthralgia in 17 out of 33%, and mild grade 1 mucositis in 22% with no ⩾grade 2. Grade 2 peripheral neuropathy was observed in 35% of patients and usually resolved to ⩽grade 1 in the majority.

### Dose intensity analysis

The administered median dose intensities for each drug of the TIP combination were as follows: for paclitaxel 52.0 mg m^−2^ week^−1^ (range: 48.3–58.3) or 89.2% (range: 82.2–100%) of the planned dose, for ifosfamide 1.5g m^−2^ week^−1^ (range: 1.2–1.67) or 89.8% (range: 71.8–100%) of the planned dose, and for cisplatin 23.0 mg m^−2^ week^−1^ (range: 19.6–26.6) or 86.7% (range: 73.7–100%) of the planned dose (Table 5). Therefore, patients received >85% of the planned dose intensity for all cytotoxic drugs in the regimen.

## Discussion

Despite significant efforts invested in clinical investigation over the past two decades, the treatment options for patients with locally recurrent/advanced after radiotherapy±surgery or *de novo* metastatic cervical cancer remain limited, and survival for these patients has remained notoriously unchanged and disappointing, with almost no long-term survivors. Cisplatin-based chemotherapeutic regimens in combination with other active drugs have resulted in both higher RRs and prolonged PFS or OS when compared with single-agent cisplatin, as verified in large phase III randomised trials (Omura *et al*, 1997; Moore *et al*, 2004; Long *et al*, 2005). However, the effect of the traditional cisplatin-based combinations in the outcome of recurrent/advanced or metastatic cervical cancer seems to be limited. Therefore, the search for newer two-drug or three-drug cisplatin-based combinations is warranted.

Paclitaxel represents an established active cytotoxic agent against advanced/metastatic cervical cancer at doses between 170 and 250 mg m^−2^ (McGuire *et al*, 1996; Kudelka *et al*, 1997). Ifosfamide represents an oxazaphosphorine alkylating agent that has yielded an 11% RR in pretreated and a 15.7% RR in chemotherapy-naive patients with advanced cervical cancer, whereas at high doses of 3.5 g m^−2^ × 5 days, a 50% RR in untreated patients was observed (Cervellino *et al*, 1990). Moreover, the doublet combinations of ifosfamide+cisplatin and paclitaxel+cisplatin (TP) have shown improved RRs and PFS over single-agent cisplatin in two phase III GOG studies (Omura *et al*, 1997; Moore *et al*, 2004).

**Figure 2:**
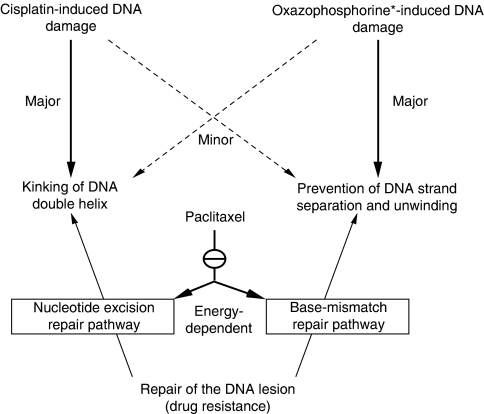
Paclitaxel inhibition of repair of the DNA damage induced by cisplatin and oxazaphosphorine ^*^(cyclophosphamide or ifosfamide) cytostatics (adapted and modified from Reed *et al*, 1995).

Preclinical data have shown that paclitaxel intensifies the cell-killing effects of chemically induced DNA damage by alkylating agents and cisplatin, provided that paclitaxel precedes these agents (Parker *et al*, 1993, Liebmann *et al*, 1994). In the clinical setting, paclitaxel has shown enhanced activity and possibly synergistic effects when combined with alkylating agents, cyclophosphamide and ifosfamide (Bunnell *et al*, 1998), or cisplatin (Rowinsky *et al*, 1993). The proposed mechanisms of *in vitro* and *in vivo* synergism are provided in Figure 2 (see also Lind *et al*, 1989; Reed *et al*, 1995).

Establishment of the recommended doses of each agent, paclitaxel, ifosfamide, and cisplatin, was based in our previous phase I study of TIP (Kosmas *et al*, 2000a); however, we elected to reduce the recommended dose of cisplatin from 100 to 80 mg m^−2^ (divided over 2 days) to compensate for imminent toxicity, particularly in patients exposed to previous pelvic radiotherapy.

The TIP regimen as applied in this study yielded efficacy and survival figures comparable with those obtained in three other published studies (Zanetta *et al*, 1999; Dimopoulos *et al*, 2002; Choi *et al*, 2006). The study by Zanetta *et al* (1999) evaluated a mixed population of 45 patients with inoperable advanced and/or metastatic cervical carcinoma, with 14 out of 45 patients having never had pelvic irradiation earlier, and 10 patients who underwent locoregional surgery with radical hysterectomy/pelvic±paraortic lymphadenectomy after a favourable response to TIP essentially having the role of neoadjuvant/induction chemotherapy. An ORR was seen in 67% of patients with a 75% RR in non-irradiated and 52% RR in previously irradiated pelvic areas. Overall survival at the time of publication for patients with CR, PR, and non-responders was 13+, 9+, and 6 months, respectively. Although the authors report a high incidence, that is, 91%, of grade 3–4 myelotoxicity, they do not provide any data on the incidence of febrile neutropenia. Moreover, prophylactic G-CSF was not routinely administered to all patients, but only as secondary prophylaxis after significant myeloid lineage toxicity, and the regimen was modified to a 50-mg m^−2^ dose of cisplatin in patients who had undergone pelvic radiotherapy earlier (*vs* 75 mg m^−2^ for non-irradiated patients) with a 24-h infusion of paclitaxel 175 mg m^−2^ and ifosfamide 5.0g m^−2^. In a more recent randomised phase II study of the same group evaluating neoadjuvant TIP *vs* TP, aiming towards radical surgery in chemotherapy- and radiotherapy-naive patients, the incidence of grade 3–4 neutropenia was 76% for TIP *vs* 26% for TP without routine prophylactic G-CSF (Lissoni *et al*, 2009).

In the study by Dimopoulos *et al* (2002), ORR was 46% (CR=19%+PR=27%) with an 11.5-month median response duration, 8.3-month median TTP, and 18.6-month median OS. It should be noted that despite the apparently adequate doses applied (paclitaxel 175 mg m^−2^, cisplatin 75 mg m^−2^, and ifosfamide 4.5g m^−2^, divided over 3 days), the regimen was less intense, as it was administered at an intended 4-week interval, contrary to the usual 3-week interval pertaining to our and the other published studies so far (Zanetta *et al*, 1999; Choi *et al*, 2006). Despite routine G-CSF prophylaxis, a 26% incidence of grade 4 neutropenia was reported.

In the more recently published study by Choi *et al* (2006), ORR was 46.7% (CR=4.4%+PR=42.2%) with a median TTP of 8.0 months and OS of 19 months. However, in this latter study, an appreciable number of patients, 17 out of 45 (38%), received TIP as second-line chemotherapy after failure of first-line cisplatin+5-FU, and this group fared significantly worse in terms of ORR and survival compared with those who received TIP as first-line chemotherapy (Choi *et al*, 2006). Drug doses in this study were lower than those in all other published studies, namely paclitaxel 135 mg m^−2^, cisplatin 50 mg m^−2^, and ifosfamide 3.0g m^−2^, divided over 3 days at 3-week intervals, justified by the authors as a result of previous pelvic irradiation in the majority of patients (96%). However, only seven episodes of febrile neutropenia were recorded (out of 253 cycles) without routine prophylactic G-CSF, and the incidence of >grade 2 peripheral neuropathy was 21%, the latter rather as a result of previous extensive systemic cisplatin chemotherapy.

Our results, with an ORR of 62% (CR=26%+PR=36%), a median TTP of 7 months, and a median OS of 16.5 months, are close to those obtained by Zanetta *et al* (1999), and compare favourably with those obtained in the other two studies (Dimopoulos *et al*, 2002; Choi *et al*, 2006). Despite a higher proportion of patients who underwent pelvic irradiation earlier (79%), compared with the Italian study (Zanetta *et al*, 1999) (69%), our results were not inferior, as the doses of drugs applied were not compromised, and this was reflected by the 21% incidence of febrile neutropenia despite routine prophylactic G-CSF. However, the majority of these febrile neutropenic episodes were managed uneventfully in the outpatient setting. Despite the limitations of inter-study comparisons, ORR were inferior in the studies by Dimopoulos *et al* (2002) and Choi *et al* (2006), presumably as a result of significant under-dosing, an arbitrarily selected lower dose intensity in the first study (4-week interval) and lower doses of drugs in the second study. In the latter study, significant under-dosing was reflected by the low incidence of grade 3–4 neutropenia, that is 11% (Choi *et al*, 2006). However, despite the higher ORR in our study, PFS and OS figures were comparable between this study and that by Dimopoulos *et al* (2002), presumably as a result of differing patient populations enrolled; more patients in this study failed within earlier radiation field, that is 60 *vs* 49%, and more patients in the study by Dimopoulos *et al* (2002) had non-squamous histologies, 17 *vs* 32%.

Results between studies may vary as a consequence of the inclusion of different proportions of patients exposed to earlier pelvic radiotherapy, differences in the PS of enrolled patients, variable inclusion of patients having failed within or outside the radiation field, doses of drugs applied, and histologic types treated (squamous vs other). Moreover, in one of the above studies, the so-called radiation-free interval (time elapsed from previous pelvic radiotherapy to recurrence, <12 *vs* ⩾12 months) seemed to have an important role in outcome after TIP chemotherapy (Zanetta *et al*, 1999).

The TIP regimen has been applied as neoadjuvant induction chemotherapy in patients with previously untreated inoperable localised cervical carcinoma by the above-referred Italian cooperative group (Zanetta *et al*, 1998). After an impressive pathological response of 34%, the investigators of SNAP (Studio Neo-Adjuvante Portio) proceeded to a phase II randomised study of neoadjuvant chemotherapy evaluating TIP *vs* the doublet of ifosfamide–cisplatin (IP), and illustrated the superiority of TIP *vs* IP with an optimal pathological response (OPR) of 48 *vs* 22%, respectively (Buda *et al*, 2005). Given the above results, a subsequent randomised study by the same group, SNAP-02, compared TIP with TP in an identical setting of neoadjuvant induction chemotherapy and confirmed once again the superior efficacy of TIP *vs* TP; OPR 43% *vs* 23%, respectively (Lissoni *et al*, 2009). There are no studies so far that have compared in a randomised manner TIP *vs* TP or IP in advanced/metastatic cervical cancer. Moreover, in general, there is a paucity of studies comparing three-drug with two-drug regimens in an advanced/metastatic setting, and only one such study has compared bleomycin–ifosfamide–cisplatin (BIP) with IP, showing no benefit for the triplet regimen, pointing rather to the lack of efficacy of bleomycin as a single agent in advanced cervical cancer (Herod *et al*, 2000). It is believed that three-drug regimens, although more efficacious than two-drug regimens at the expense of increased toxicity, do not ultimately prolong PFS or OS in advanced/metastatic disease. In this setting, the TP doublet has shown to prolong median PFS over single-agent cisplatin (Moore *et al*, 2004), and the topotecan–cisplatin two-drug regimen has yielded improved median PFS and OS (Long *et al*, 2005).

With increasing numbers of patients who receive concurrent chemoradiotherapy with cisplatin as standard primary treatment for locally advanced cervix cancer since 1999–2000, when the combination emerged as standard, the RR, PFS, and OS rates for single-agent cisplatin have declined in relapsing patients, and a relative cisplatin resistance may explain the differences seen in recent phase III trials comparing combination chemotherapy with single-agent cisplatin. However, as explained theoretically above, the TIP combination may overcome resistance to cisplatin at least partially. A recent GOG study (GOG-204) – the largest phase III randomised trial in recurrent/metastatic cervical cancer – compared four platinum-based doublets with the reference arm of paclitaxel–cisplatin, which was found to be not inferior to the three investigational arms (Monk *et al*, 2008). Moreover, another GOG phase II study (GOG-227C) of targeted anti-vascular therapy (bevacizumab 15 mg kg^−1^) in this patient population showed activity comparable with cytotoxics and was well tolerated (Monk *et al*, 2009).

Our results highlight the important activity of TIP combination in relapsed/metastatic cervical cancer, even in cases with recurrences within previously irradiated pelvis, however, at the cost of increased but manageable haematologic toxicity, and point to the design of randomised phase III studies comparing TIP with doublets in appropriately selected patients with advanced/metastatic cervical cancer.

## Figures and Tables

**Table 1:** Patient characteristics

**Characteristic**	**No.**	**%**
Number of patients	42	100
		
*Age (years)*		
Median (range)	56 (25–74)	
		
*Performance status (WHO)*		
0	23	55
1	16	38
2	3	7
		
*Histology*		
Squamous	35	83
Adenosquamous	5	12
Adenocarcinoma	2	5
		
*Previous treatment*		
Surgery alone	2	4
Surgery → radiation	5	12
Surgery → chemoradiation	10	24
Concurrent chemoradiation	18	43
None	7	17
		
*Sites of tumour involvement*		
Within prior radiation field (pelvis)	18	43
Outside prior radiation field	17	40
Both	7	17
		
*Metastatic sites*		
Lymph nodes	36	86
Pelvic mass	26	62
Lung	12	29
Liver	3	7
Bone	2	4
		
*No. of sites with tumour involvement*		
1	15	36
2	23	55
>3	4	9

Abbreviation: WHO=World Health Organization.

**Table 2:** Response to TIP regimen by disease site

	**All patients**	**Within irradiated pelvis**	**Outside irradiated pelvis**
**Response**	**No. (%)**	**No. (%)**	**No. (%)**
CR	11 (26)	1 (4)	10 (42)
PR	15 (36)	7 (28)	8 (33)
SD	11 (26)	12 (48)	6 (25)
PD	5 (12)	5 (20)	0 (0)
Total	42 (100)	25 (100)	24 (100)

Abbreviations: CR=complete response; PD=progressive disease; PR=partial response; SD=stable disease; TIP=paclitaxel–ifosfamide–cisplatin.

**Table 3:** Haematologic toxicities (WHO grade) for TIP

	**WHO grade (% of patients, all cycles)**				
**Toxicity**	**0**	**1**	**2**	**3**	**4**
Leukopenia	0	5	11	30	54
Neutropenia	0	4	13	26	57
Thrombocytopenia	15	48	28	7	2
Anaemia	45	24	24	7	0
Febrile Neutropenia	21%				

Abbreviations: TIP=paclitaxel–ifosfamide–cisplatin; WHO=World Health Organization.

**Table 4:** Non-haematologic toxicities (WHO grade) for TIP

	**WHO grade (% of patients, all cycles)**				
**Toxicity**	**0**	**1**	**2**	**3**	**4**
Nausea and vomiting	24	41	13	22	0
Mucositis	72	28	0	0	0
Myalgia/arthralgia	50	17	33	0	—
Neurologic					
Peripheral	14	51	35	0	0
CNS	74	24	2	0	0
Infection	93	5	0	2	0
Diarrhoea	51	18	31	0	—
Allergy	93	7	0	0	0
Alopecia	0	0	15	85	0
Asthenia/fatigue	16	38	31	15	—
Cardiac	98	2	0	0	0
Renal	96	2	2	0	0
Haematuria	98	2	0	0	0
Electrolyte^a^	95	5	0	0	0

Abbreviations: CNS=central nervous system; TIP=paclitaxel–ifosfamide–cisplatin; WHO=World Health Organization.

a Electrolyte toxicity refers to serum potassium (K^+^) and magnesium (Mg^2+^) drop.

**Table 5:** Dose intensity analysis of TIP in cervical cancer

**Drug**	**Planned dose (every 3 weeks)**	**Actual mean DI (mg** **m**^−**2**^ **week**^**−1**^**) (range)**	**Planned DI** (**mg** **m**^−**2**^ **week**^**−1**^)	**% of planned DI**
Paclitaxel	175 mg m^−2^	52.0 (48.3–58.3)	58.3	89.2 (82.2–100)
Ifosfamide	5.0 g m^−2^	1.50 (1.2–1.67)	1.67	89.8 (71.8–100)
Cisplatin	80 mg m^−^^2^	23.0 (19.6–26.6)	26.6	86.7 (73.7–100)

Abbreviations: DI=dose intensity; TIP=paclitaxel–ifosfamide–cisplatin.
